# Consensus on the therapeutic management of atopic dermatitis ‒ Brazilian Society of Dermatology: an update on phototherapy and systemic therapy using e-Delphi technique^☆^

**DOI:** 10.1016/j.abd.2023.04.003

**Published:** 2023-06-09

**Authors:** Raquel Leao Orfali, Daniel Lorenzini, Aline Bressan, Anber Ancel Tanaka, Ana Maria Mósca de Cerqueira, André da Silva Hirayama, Andréa Machado Coelho Ramos, Carolina Contin Proença, Claudia Marcia de Resende Silva, Cristina Marta Maria Laczynski, Francisca Regina Carneiro, Gleison Duarte, Gunter Hans Filho, Heitor de Sá Gonçalves, Ligia Pessoa de Melo, Luna Azulay-Abulafia, Magda Blessmann Weber, Maria Cecília Rivitti-Machado, Mariana Colombini Zaniboni, Marília Ogawa, Mario Cezar Pires, Mayra Ianhez, Paulo Antonio Oldani Felix, Renan Bonamigo, Roberto Takaoka, Rosana Lazzarini, Silmara Cestari, Silvia Assumpção Soutto Mayor, Tania Cestari, Zilda Najjar Prado de Oliveira, Phyllis I. Spuls, Louise A.A. Gerbens, Valeria Aoki

**Affiliations:** a Department of Dermatology, Faculdade de Medicina, Universidade de São Paulo, São Paulo, SP, Brazil; b Department of Dermatology, Irmandade Santa Casa de Misericórdia de Porto Alegre, Porto Alegre, RS, Brazil; c Department of Dermatology, Hospital Universitário Pedro Ernesto, Universidade Estadual do Rio de Janeiro, Rio de Janeiro, RJ, Brazil; d Department of Dermatology, Hospital Universitário Evangélico Mackenzie, Curitiba, PR, Brazil; e Department of Dermatology, Hospital Municipal Jesus, Rio de Janeiro, RJ, Brazil; f Department of Dermatology, Hospital das Clínicas, Faculdade de Medicina, Universidade de São Paulo, São Paulo, SP, Brazil; g Department of Dermatology, Hospital das Clínicas, Universidade Federal de Minas Gerais, Belo Horizonte, MG, Brazil; h Dermatology Clinic, Irmandade Santa Casa de Misericórdia de São Paulo, São Paulo, SP, Brazil; i Department of Dermatology, Centro Universitário Faculdade de Medicina do ABC, Santo André, SP, Brazil; j Department of Dermatology, Universidade do Estado Pará, Belém, PA, Brazil; k Department of Dermatology, Instituto Bahiano de Imunoterapia, Salvador, BH, Brazil; l Department of Dermatology, Hospital Universitário Maria Aparecida Pedrossian, Universidade Federal de Mato Grosso do Sul, Campo Grande, MS, Brazil; m Department of Health, National Reference Center in Sanitary Dermatology Dona Libânia, Fortaleza, CE, Brazil; n Department of Dermatology, Instituto de Medicina Integral Professor Fernando Figueira, Recife, PE, Brazil; o Health Department, Hospital Otávio de Freitas, Recife, PE, Brazil; p Professor Rubem David Azulay Institute of Dermatology, Santa Casa de Misericórdia do Rio de Janeiro, Rio de Janeiro, RJ, Brazil; q Universidade Federal de Ciências da Saúde de Porto Alegre, Porto Alegre, RS, Brazil; r Department of Dermatology, Universidade Metropolitana de Santos, Santos, SP, Brazil; s Department of Dermatology, Universidade Federal de São Paulo, São Paulo, SP, Brazil; t Department of Dermatology, Complexo Hospitalar Padre Bento, Guarulhos, SP, Brazil; u Department of Dermatology, State Public Servant Hospital, São Paulo, SP, Brazil; v Department of Dermatology, Hospital for Tropical Diseases, Goiânia, GO, Brazil; w Department of Dermatology, Universidade Federal de Goiás, Goiânia, GO, Brazil; x Department of Dermatology, Federal Hospital of State Servers, Rio de Janeiro, RJ, Brazil; y Department of Dermatology, Universidade Federal do Rio Grande do Sul, Porto Alegre, RS, Brazil; z Department of Dermatology, Teaching and Research Institute of Hospital Sírio-Libanês, São Paulo, SP, Brazil; A Department of Dermatology, Amsterdam UMC, Academic Medical Center, University of Amsterdam, Amsterdam Public Health, Infection and Immunity, The Netherlands

**Keywords:** Atopic dermatitis, Delphi technique, Medication therapy management, Phototherapy

## Abstract

This publication is an update of the “Consensus on the therapeutic management of atopic dermatitis – Brazilian Society of Dermatology” published in 2019, considering the novel, targeted-oriented systemic therapies for atopic dermatitis. The initial recommendations of the current consensus for systemic treatment of patients with atopic dermatitis were based on a recent review of scientific published data and a consensus was reached after voting. The Brazilian Society of Dermatology invited 31 experts from all regions of Brazil and 2 international experts on atopic dermatitis who fully contributed to the process. The methods included an e-Delphi study to avoid bias, a literature search and a final consensus meeting. The authors added novel approved drugs in Brazil and the indication for phototherapy and systemic therapy for AD. The therapeutical response to systemic treatment is hereby reported in a suitable form for clinical practice and is also part of this updated manuscript.

## Introduction

For many years, the dermatology scope of conventional systemic therapy to treat moderate-to-severe AD was limited to ciclosporin, methotrexate, azathioprine, mycophenolate mofetil and systemic glucocorticoids, with scarce evidence data. Systemic therapy for AD is recommended when the disease lacks control after topical treatment with anti-inflammatory drugs, associated with basic steps such as the identification of triggers, educational programs, and phototherapy. In this context, the patients and caretakers preferences and accessibility must be taken into account, evaluating the AD impact on their personal life, financial implications, and comorbidities.^1^, ^2^, ^3^, ^4^

Considering the current developments in the systemic therapy of atopic dermatitis (AD) with targeted-oriented therapies, the inclusion of newly approved drugs and updates concerning the indication for conventional systemic therapy, this study became necessary. The aim of this Brazilian consensus, therefore, is to provide guidance and recommendations on the management of phototherapy and systemic therapy for moderate to severe AD in adult and pediatric patients, and also to endorse the HOME (Harmonizing Outcome Measures for Eczema), the Core Outcome Set (COS) and corresponding core outcome instruments to unify outcome reporting in clinical trials and clinical practice.^5^, ^6^

## Methods

The Delphi technique is a recognized method used to gain consensus among specialists in a particular field, where expert opinion is important in defining judgments. This approach provides experts with an opportunity to alter their responses based on their peers experiences, thus increasing the likelihood of convergence of opinion.^7^, ^8^ The process is anonymous to avoid participants being influenced by the opinions of their peers and to avoid response bias.^7^, ^8^ The e-Delphi technique is an adaptation of the Delphi methodology with the inclusion of internet-based research which allows the expansion of the use of the benefits and reduces the limitations of the traditional Delphi method.^9^

In the current consensus study, the process consisted of online questionnaires (based on a review of recent literature), followed by an online consensus meeting, where final disagreements were solved by voting, concluding with a validation stage of the written consensus via e-mail.

### Scope of the intended consensus

Update on the management of phototherapy and systemic therapy for moderate to severe AD (in adults and children)^10^ in Brazil.

### Terminology used

The recommendations used for the consensus study (evidence and treatment goals) were based on the updated consensus on systemic therapy for AD and the definitions of treatment goals. The evidence for treatment recommendations were based on EDF (European Dermatology Forum) guidelines^11^, ^12^; Treat-to-Target in AD by De Bruin-Weller et al.,^13^ NMA analysis (Systematic Review and Network Meta-analysis) by Drucker et al.^4^, ^14^; Schünemann et al.^15^; Guyatt et al.^16^; HOME initiative,^5^ and International Eczema Council (IEC) initiative.^17^

### Wording of recommendations

To standardize the wording of recommendations used in this study was the same used by the GRADE group,^15^, ^16^ and Living EuroGuiDerm Guideline for the systemic treatment of Atopic Eczema, 11, 12 described as: “We recommend” (Strong recommendation for using an intervention); “We suggest” (Weak recommendation for using an intervention); “We suggest against” (Weak recommendation against using an intervention); “We recommend against” (Strong recommendation against using an intervention). The strength of the recommendation is determined by the balance between desirable and undesirable consequences of alternative management strategies, quality of evidence, variability in values, and individualized preferences.^15^, ^16^

### Participants

Thirty-one dermatologists selected by the Brazilian Society of Dermatology (BSD), and two international experts on AD (with high expertise in systematic reviews, international guidelines, and consensus exercises), participated in the consensus study. The inclusion criteria for the participants were board certification (BSD) with expertise in AD, and/or in the academic field, representing different regions of the country.

Since the study was not part of any medical research involving human subjects, there was no need for approval by ethics committees.

### E-Delphi questionnaire

RLO, VA, DL, PS, and LG designed and reviewed the questionnaire, based on current scientific data and literature review,^4^, ^5^, ^11^, ^12^, ^13^, ^14^, ^15^, ^16^ attempting to fulfill and update gaps of the last published consensus.^10^ The questionnaire had 31 questions (Table 1, Table 2, Table 3, Table 4, Table 5), divided into four domains: Implementation of the international consensus-based Core Outcome Set (COS) by the Harmonising Outcomes Measures for Eczema (HOME) initiative^5^; Selecting systemic treatment modalities for individual AD patients; Need for changes in the follow up of systemic therapy based on treat-to-target goals of international eczema council^13^, ^17^; and Introduction of systemic target-oriented therapies for adults and children with moderate to severe AD.

**Table 1:** BSD experts position regarding AD diagnostic criteria, Core Outcome Sets (COS) for clinical practice and for clinical trials

Outcome	BSD experts’ votes
The diagnosis of AD will be defined according to clinical criteria (Hanifin & Rajka^18^ or UK working party's diagnostic criteria)^20^	
For clinical practice, the recommended instruments for assessing patient-reported symptoms are: PO-SCORAD; POEM; NRS-11 in past 24h; PROMIS® Itch Questionnaire – average 1-week NRS itch and peak 1-week NRS itch^5^	
For clinical practice, the recommended instruments for evaluating long-term disease control are: ADCT, RECAP, and PGA^5^	
The following core outcome domains and instruments should be measured and reported in ALL AD trials: Clinician-reported signs – EASI; Patient-reported symptoms: POEM and NRS-11 in past 24h; Quality of Life - DLQI (adults), CDLQI (children), IDQoL (infants); Long-term control - RECAP or ADCT^5^	

AD, Atopic Dermatitis; PO-SCORAD, Patient-Oriented SCORing Atopic Dermatitis; POEM, Patient Oriented Eczema Measure; NRS-11, Peak Pruritus Numerical Rating Scale; PROMIS, Patient-Reported Outcomes Information System; ADCT, Atopic Dermatitis Control Tool; RECAP, Instrument (seven-item questionnaire) to Capture the patient perspective of eczema control; PGA, Patient Global Assessment; EASI, Eczema Area and Severity Index; DLQI, Dermatology Life Quality Index; CDLQI, Children Dermatology Life Quality Index; IDQol, Infants' Dermatitis Quality of Life Index.

**Table 2:** BSD experts position regarding selecting systemic treatment modalities for individual AD patients

Outcome	BSD experts’ votes
When selecting systemic treatment modalities for individual AD patients; it is relevant to ask the medical history for comorbidities	
Physicians should be aware of AD associated atopic and non-atopic comorbidities when considering treatment options	
Start of a therapeutic option (topical; phototherapy or systemic therapy) in AD should be a shared decision between patients; caretakers; and physicians	
Systemic therapy for AD is recommended when the disease lacks control after topical treatment with anti-inflammatory drugs; removing triggers; educational programs (when needed); phototherapy. Needs to be considered: respecting patients’ preferences and evaluating AD impact on personal life, financial implications, and comorbidities	

AD, Atopic Dermatitis.

**Table 3:** BSD experts position regarding phototherapy and conventional systemic therapy for AD

Outcome	BSD experts’ votes
Phototherapy may be indicated as an alternative treatment for moderate-to-severe and refractory to topical treatment AD	
NB-UVB and UVA1 are considered useful phototherapies in patients with moderate-to-severe AD	
Short-term treatment with oral glucocorticoids may be considered for adults and in exceptional cases also for children and adolescents with severe AD to control flares and to prepare them for other subsequent therapeutic modalities after discontinuation of a previous systemic therapeutic option. Exceptional cases include: lack of other treatment options; as a bridge to other systemic therapies or phototherapy; during acute flares in need of immediate relief; in anticipation of a major life event; or in the most severe and recalcitrant cases	
Systemic glucocorticoids treatment should be limited to short-term use with a long-term plan for therapy using non-steroid systemic drugs	
Ciclosporin (CsA) may be considered a systemic option for severe AD (licensed in Brazil) when it is not possible to taper potent topical steroids	
CsA initial dose varies from 2.5 to 5.0 mg/kg/day. Usage of higher initial dose of 4-5 mg/kg/day may lead to a more rapid response	
The recommended length of treatment with CSA for patients with AD is up to 1 year	
CsA as a systemic treatment for AD may be extended for a period above 1 year, if well tolerated	
Tapering CsA is possible after achievement of marked clinical improvement in AD; with recommended reduction of 0.5-1.0 mg/kg/day every two weeks	
The expected risk-benefit ratio needs to be evaluated before using CsA in AD patients, in comparison with other systemic therapeutic alternatives	
Monitoring blood pressure levels and renal function are recommended before initiation and during follow-up in AD patients on CsA	
After extensive UV therapy, CsA usage is not ideal due to the increased risk of skin cancer	
MTX may be considered as a long-term systemic treatment for moderate-to-severe AD when it is not possible to taper potent topical steroids (off-label in Brazil)	

AD, Atopic Dermatitis; NB-UVB, Narrow-band Ultraviolet B; UVA-1, Ultraviolet A-1; CsA, Ciclosporin; MTX, Methotrexate.

**Table 4:** BSD experts position regarding the need for changes in systemic therapy decision for AD patients

Outcome	BSD experts’ votes
When considering the need for changes in systemic therapy; two decision-points should be considered to check whether the optimal treatment target was reached (after 3 and 6 months). Each decision-point should be based on improvements in PGA plus at least one specific clinical outcome domain^13^	
The initial acceptable treatment target goals after 3 months should reach: at least EASI 50 or SCORAD 50 (50% reduction compared to baseline); For Peak Pruritus NRS (0–10): a reduction of at least 3 points; For DLQI: a reduction of at least 4 points; For POEM: a reduction of at least 4 points^13^	
The optimal acceptable treatment target goals after 6 months should reach at least: EASI 75 or EASI ≤ 7; SCORAD 75 (75% reduction compared to baseline) or SCORAD ≤ 24; Peak Pruritus NRS (0–10): an absolute score ≤ 4; DLQI: an absolute score ≤ 5; POEM: an absolute score ≤ 7^13^	
The initial acceptable treatment target goal after 3 months should reach a reduction of at least 1 point in patient global response (e.g., PtGA 0-4)^13^	
The optimal acceptable treatment target goal according to PGA (e.g., PtGA 0-4) after 6 months should reach an absolute score of ≤ 2,^13^ but if target outcomes are achieved for PGA plus at least 1 specific disease domain (signs, symptoms, quality of life), treatment continuation should be considered	
Changes in systemic therapy may be needed in the presence of specific or non-specific undesirable adverse events (e.g. infection) occur under pharmacotherapy; or when there is a contraindication to continuing therapy (e.g., desire to have children; becoming pregnancy)	

AD, Atopic Dermatitis; SCORAD, SCORing Atopic Dermatitis; POEM, Patient Oriented Eczema Measure; NRS-11, Peak Pruritus Numerical Rating Scale; PGA, Patient Global Assessment; DLQI, Dermatology Life Quality Index; EASI, Eczema Area and Severity Index; PtGA, Patient self-reported Global Assessment of disease severity.

**Table 5:** BSD experts position regarding the introduction of systemic target-oriented therapies for adults and children with moderate-to-severe AD

Outcome	BSD experts’ votes
If patient is not well controlled with conventional therapies (e.g., phototherapy; CsA or MTX) introduction of systemic target-oriented therapies for adults and children approved for moderate-to-severe AD such as immunobiologics and/or Janus-kinase inhibitors should be considered; endorsed by any of the conditions above except for child wish; lactating; and pregnancy	
Dupilumab should be a systemic target-oriented option for adults with AD (initially 600 mg SC day 1 followed by 300 mg Q2W); and adolescents / children (age 12-17: < 60 kg: initially 400 mg SC day 1 followed by 200 mg Q2W; when ≥ 60 kg: initially 600 mg SC day 1 followed by 300 mg Q2W); age 6-11: from 15kg to < 60 kg; initially 600 mg SC day 1 followed by 300 mg Q4W; when ≥ 60 kg; initially 600 mg SC day 1 followed by 300 mg Q2W; age 6 months-5 years: 15 to < 30 kg, initial and subsequent dosage of 300 mg Q4W, 1 x 300 mg; 5 to < 15 kg, initial and subsequent dosage 200 mg Q4W, 1 x 200 mg). (Licensed in Brazil)	
Baricitinib should be a systemic target-oriented option for adults with AD (4 mg per day; reduction to 2 mg per day possible; depending on treatment response). (Licensed in Brazil)	
Upadacitinib should be a systemic target-oriented option for adults: 15 or 30mg/day; age ≥ 65: 15mg/day and adolescents with AD (age 12-17 > = 30 kg bw: 15 mg per day). (Licensed in Brazil; in license for ≤ 12 years)	

AD, Atopic Dermatitis; CsA, Ciclosporin; MTX, Methotrexate; SC, Subcutaneous; Q2W, Every other week; Q4W, Every 4 weeks.

### E-Delphi study

The e-Delphi study was performed in 2 rounds. In round 1 the participants voted on the proposed subjects. Those statements that met the agreement criterium (above 75% of agreement) were endorsed and were not available for voting in subsequent e-Delphi rounds. Those statements that did not reach at least 75% of agreement, were reviewed by BSD members and resubmitted for a new e-Delphi voting round, when the participants were able to view the voting results for the previous e-Delphi round (in accordance with anonymity and randomization of the Delphi study, to avoid bias). The possible answers were scored on a 1‒9 Likert scale (1‒3: Not important; 4‒6: important but not critical; 7‒9: Critical, and an unable to rate option), using the Delphi Manager v5.0 platform (https://delphimanager.liv.ac.uk), with selection of one single choice. Consensus for all given statements required 75% or more of all participants to rate their level of agreement as 7, 8, or 9 according to the Likert scale. For data analysis and graphics generation, the authors applied GraphPad Prism v9.1.4 (GraphPad Software, San Diego, CA, USA).

### Consensus meeting and validation of written consensus

After round 2, BSD experts participated in on-line consensus meetings. They endorsed the statements that had reached consensus in the e-Delphi rounds and discussed the ones that had not reached consensus. As described above, consensus required at least 75% of agreement on any given statement. All BSD experts participated in the online meetings. Finally, the consensus statements including the literature review were compiled into the updated Brazilian consensus paper for phototherapy and AD systemic treatment and validated by email.

## Results

The e-Delphi consensus study connecting a group of Brazilian physicians, from different regions of the country, with established experience in treating patients with moderate-to-severe AD, easily reached an agreement on a single set of statements related to moderate-to-severe AD requiring systemic therapy.

### Implementation of diagnostic criteria and consensus-based core outcome set (COS) of the HOME initiative

The diagnosis of AD is based on clinical findings according to the Hanifin & Rajka diagnostic criteria,^18^ described in the 1980’s and widely recognized.^19^ In 1997, the U.K. working group diagnostic criteria for AD were introduced by Williams et al.^20^ as a refinement of Hanifin and Rajka’s diagnostic criteria for AD. A systematic review comparing the validation of various diagnostic criteria for AD concluded that the U.K. working group diagnostic criteria is the most validated.^19^

The international HOME initiative (www.homeforeczema.org) established a Core Outcome Set (COS) for clinical trials and a clinical practice set to unify outcome reporting.^6^, ^21^ The clinical trials set included clinician-reported signs – EASI-eczema area and severity index (range from 0‒72, where a score of 0 indicates clear or no eczema; 0.1 to 1.0 indicates almost clear; 1.1 to 7 indicates mild disease; 7.1 to 21 indicates moderate disease; 21.1 to 50 indicates severe disease; and >51 indicates very severe disease); patient-reported symptoms – POEM (Patient-Oriented Eczema Measure) and NRS-11 (peak pruritus numerical rating scale) in the past 24 h; quality of life ‒ DLQI (adult Dermatology Life Quality Index), CDLQI (children Dermatology Life Quality Index), IDQoL (infants Dermatitis Quality of Life Index); long-term control – RECAP (instrument (seven-item questionnaire) to capture the patient perspective of eczema control) or ADCT (atopic dermatitis control tool). The clinical practice set comprised, for assessing patient-reported symptoms, PO-SCORAD (patient-oriented SCORing atopic dermatitis); POEM; NRS-11 in the past 24 h; PROMIS® itch questionnaire – average 1-week NRS itch and peak 1-week NRS itch; and for evaluating long-term disease control ADCT, RECAP, and PGA (patient global assessment).^6^, ^21^ Both sets are based on extensive literature reviews and consensus meetings, in which different groups participated from all parts of the world.

#### Consensus

In this consensus, the authors discussed both diagnostic criteria and BSD experts agreed (93.5% of participants) to recommend and support both established criteria. BSD experts recommended and supported the COS usage recognized by the HOME initiative (80% are related to instruments recommended to assess patient-reported symptoms and 75.8% to instruments to assess long-term disease control), and 92.8% for clinical trials (Table 1).

### Selecting of systemic treatment modalities for individual AD patients

One of the indications for systemic treatment in patients with moderate-to-severe AD includes failure to respond to topical therapies. Before initiating systemic treatment, it is mandatory to avoid aggravating factors, to diagnose and treat secondary infections, and rule out differential diagnoses. The option for systemic therapy should also include the impact of the disease on patients quality of life and a careful assessment of risks and benefits of the chosen medication and should be a consensual decision of the patient and the physician.^10^, ^11^, ^13^, ^17^ The choice of systemic therapy should take into consideration the age of patients children, infants, adolescents, adults, and elderly with associated comorbidities.^3^, ^13^, ^22^, ^23^, ^24^, ^25^

Conventional systemic therapy options for AD include ciclosporin (CsA), methotrexate (MTX), azathioprine (AZA), mycophenolate mofetil (MMF), and systemic glucocorticoids and were discussed in the past Brazilian consensus.^10^ In the present consensus, the authors updated recommendations regarding CSA, MTX and systemic glucocorticoids. The recommendations for AZA and MMF remain as stated in the last consensus^10^ and will not be discussed in this publication due to the absence of recent updates related to these drugs.^2^, ^4^, ^11^, ^26^

In the face of new developments in systemic drugs for AD, conventional therapeutic options, phototherapy, and indications had to be reconsidered and adapted to an individualized therapy strategy for non-responders to conventional and/or topical treatment, or phototherapy, focusing on systemic treatment continuation, modification, or discontinuation.^1^, ^13^

#### Consensus

All BSD experts recommend that when selecting systemic treatment modalities for individual AD patients, it is relevant to evaluate the medical history for comorbidities (as well as pregnancy, childbirth intention, and age) to detect AD-associated atopic and non-atopic comorbidities when considering treatment options, and to have a consensual decision among patients, caretakers, and physicians. Also, there is agreement that systemic therapy for AD is recommended when there is refractory disease despite adequate adherence to topical treatment with anti-inflammatory drugs, removal of triggers, educational programs, phototherapy, or when there is poor quality of life due to the impact of the disease, financial implications, and the presence of comorbidities (Table 2).

## Conventional therapeutical options for AD

### Phototherapy

#### Mechanism of action

Phototherapy is an adjuvant therapeutic option, especially for sub-acute to chronic forms of AD. It is helpful for controlling pruritus and improving the skin condition of patients who do not respond to topical medications and moisturizers.^27^, ^28^ In chronic AD, it controls clinical signs, reduces bacterial colonization by *Staphylococcus aureus* (*S. aureus*) and *Pityrosporum orbiculare,* and improves insomnia, being a steroid-sparing measure as well.^27^, ^29^, ^30^

Different wave lengths can be used to treat AD: ultraviolet B (UVB), narrow-band UVB (NB-UVB), excimer laser/light (308 nm), ultraviolet A-1 (UVA-1 340‒400 nm) in high and medium doses, and the combination of psoralens and UVA (PUVA).^31^, ^32^, ^33^, ^34^ UVB-NB (311‒313 nm) is the most widely used modality and can be indicated for children and pregnant women.^35^, ^36^ UVA-1 is seldom available in Brazil but is useful for low-responsive cases and to control flares.^12^, ^27^, ^37^

#### Safety

Evidence of phototherapy safety in AD patients is modest because there is no data from RCTs or registries with large patient cohorts or long-term follow-ups.^11^

Phototherapy acute adverse events are related to elevated temperature, stinging, erythema, and burns. However, the most relevant detrimental effects of phototherapy are those caused by chronic UV exposure such as lentigos, actinic keratosis, photoaging, and skin cancer.^38^ However, follow-up of patients submitted to NB-UVB failed to show an increased occurrence of non-melanoma skin cancer compared to the general population.^12^, ^37^ There are no long-term follow-up studies related to the induction of malignancies after UVA-1 treatments.^32^

In patients under systemic immunosuppressants (e.g., CsA and azathioprine), phototherapy is not recommended based on their risk of co-carcinogenicity.^11^, ^39^ It is also important to avoid phototherapy in patients at risk of recurrent herpes simplex infection or with a history of eczema herpeticum.^10^ The combination of NB-UVB with topical corticosteroids or topical calcineurin inhibitors is considered safe.^11^

#### Evidence

Phototherapy indications for AD treatment are largely empiric and based on reasonably few evidence-based data.^1^ A systematic review included 19 RCTs and suggested that both NB-UVB and UVA1 are the most effective phototherapy options in the treatment of AD patients.^27^ A recent study investigating itch response in patients with AD treated with NB-UVB and CsA showed that NB-UVB reduces itch significantly better than CsA.^28^

A Cochrane review assessing the effects of phototherapy for treating AD included 32 trials with 1219 randomized participants. The results indicated that when compared to placebo or no treatment, NB-UVB may improve physician-rated signs, patient-reported symptoms, and IGA (investigator global assessment) after 12 weeks of treatment, without a difference in withdrawal due to adverse events. The level of evidence for UVA1 compared to NB-UVB or PUVA, and NB-UVB compared to PUVA was very low.^40^

#### Monitoring

The BSD phototherapy practical guide recommends that ophthalmological evaluation is indicated before treatment and every six months, with an additional investigation of antinuclear antibodies (ANA) and anti-Ro (SS-A) before starting treatment in patients with a history of photosensitivity.^41^

#### Consensus

A total of 83.8% of the Brazilian experts recommended that phototherapy can be an alternative treatment for moderate-to-severe and refractory to topical treatment AD, and 80.6% validated and recommended that NB-UVB and UVA-1 are beneficial phototherapies in patients with moderate-to-severe AD (Table 3).

### Systemic glucocorticoids

#### Mechanism of action

Glucocorticoids are steroid hormones that exert an important anti-inflammatory effect through cellular and molecular mechanisms. The binding of the glucocorticoid to its receptor in the cell cytoplasm results in the activation of the receptor-glucocorticoid complex, which in turn, regulates the genes related to cytokine expression and cellular apoptosis.^42^ The proteins resulting from this activation induce the glucocorticoid response, which can be either stimulating or inhibitory, depending on the specific gene and affected tissue Thus, negative effects on gene expression contribute to the anti-inflammatory and immunosuppressive response of glucocorticoids.^43^, ^44^

#### Safety

Systemic therapy with corticosteroids, especially in high doses and prolonged use may lead to multiple side effects: suppression of growth in children, osteoporosis, adrenal insufficiency, Cushing's syndrome, hypertension, diabetes, gastritis, changes in behavior, opportunistic infections, glaucoma, cataracts, hyperlipidemia, thrombosis, and sleep disorders, among others.^45^

#### Evidence

Despite the frequent use of systemic corticosteroids in the treatment of AD and the short-term improvement in clinical signs and symptoms, there are very few randomized controlled trials, as well as clear evidence, supporting their frequent use in AD.^45^, ^46^ In addition to the side effects caused by prolonged use of systemic corticosteroids, the rebound phenomenon must be considered, i.e., the marked worsening of the disease after discontinuation of therapy.^11^, ^43^, ^47^ Recent papers, including a systematic review (Cochrane), among others, do not yield a clear indication of the use of systemic corticosteroids in AD.^48^, ^49^

#### Monitoring

Considering this context and recognizing the chronicity of the disease, the use of systemic corticosteroids should occupy only a small space in the treatment of AD. Systemic corticosteroids are recommended in short-term therapy as a quick rescue medication or serve as a bridge for the introduction of other therapies. Long-term use should be avoided, and even contraindicated, as it does not provide a stable and safe remission of AD, with the assessment of risk versus benefits being very unfavorable for the patient.^45^, ^47^

#### Consensus

BSD experts suggest that short-term treatment with oral glucocorticoids may be considered for adults and in exceptional cases, also for children and adolescents with severe AD to control flares and to prepare them for other subsequent therapeutic modalities after discontinuation, along with a long-term plan for therapy using non-steroid systemic drugs. Exceptional cases include lack of other treatment options; bridge to other systemic therapies or phototherapy; during acute flares in need of immediate relief; in anticipation of a major life event; or in the most severe and resistant AD cases (Table 3).

### Ciclosporin

#### Mechanism of action

Ciclosporin A (CsA) is a potent immunosuppressant, as it inhibits the synthesis of IL-2, essential for the anticipation and activation of lymphocytes, through the attraction of the calcineurin pathway.^50^, ^51^ It is currently considered a first-line treatment option for patients with severe AD, regardless of age.^11^

The initial dose is 5 mg/kg/day, divided into two doses for up to 6 weeks or until adequate AD control. During maintenance, it is reduced to 2.5 to 3 mg/kg/day, during three to 12 months.^11^ If necessary, the use of ciclosporin can be extended beyond one year when the patient's clinical condition allows it.^52^ Progressive withdrawal is recommended (0.5 to 1.0 mg decrease every two weeks).^50^ Another approach to treatment is the intermittent use of CsA.^2^, ^53^

#### Safety

CsA has a plasma half-life of 24 hours, hepatic metabolism, and biliary elimination,^54^ and accumulates in tissues in concentrations three to four times higher than in plasma; it remains in the lymph-myeloid tissue and fat tissue after drug suspension.^50^, ^55^, ^56^ The most common and serious adverse effect is nephrotoxicity, followed by hepatotoxicity, hypertension, anorexia, lethargy, hirsutism, tremors, paresthesia, gingival hypertrophy, and gastrointestinal disturbances, with no bone marrow depressant effect associated.^50^, ^55^

#### Evidence

A meta-analysis of 39 randomized clinical trials evaluated 6360 patients examining 20 medications and placebo comparing the effectiveness and safety of systemic immunomodulatory treatments for patients with AD. The results indicated that dupilumab and CsA were similarly effective for adult patients with AD for up to 16 weeks of treatment and more effective than MTX and AZA. More clinical trials are necessary to establish a long-term follow-up longer than 16 weeks.^14^ Although new treatment modalities such as dupilumab demonstrate better safety profiles, lower costs, and more availability are some reasons to improve the evidence profile of conventional systemic therapies like CsA.^1^

#### Monitoring

As a first step, physicians should request complete blood count evaluation, and laboratory tests for evaluating kidney and liver functions, at baseline and after the first four weeks of therapy, and then every three months. Screening for hepatitis B/C and HIV is recommended, and any signs of high blood pressure or elevated creatinine serum levels require dose reduction and monitoring.^50^ Nephrotoxicity is more frequent in the elderly, at doses greater than 5 mg/kg/day, after prolonged use, and in those with increased serum creatinine levels.^55^ Contraindications are the concomitant use of cyclosporine with phototherapy (increased risk of skin cancer) and attenuated live virus vaccines.^50^ Ciclosporin should not be used as a substitute for topical treatment.^50^ It is allowed in pregnancy, with a certain risk of low weight at birth, without risk of malformation or fetal death.^57^

#### Approval

Ciclosporin is licensed in Brazil for children ≥1 year and adults with moderate-to-severe AD.^58^

#### Consensus

BSD experts recommend that CsA may be a systemic option for severe AD, and tapering ciclosporin is possible after the achievement of marked clinical improvement in AD, with recommended reduction of 0.5‒1.0 mg/kg/day every two weeks. They also recommend that the expected risk-benefit ratio needs to be evaluated before introducing CsA in AD patients, in comparison with other systemic therapeutic alternatives. BSD experts suggest that the length of treatment with ciclosporin for patients with AD is up to 1 year but may be extended for a period above 1 year, if well tolerated (Table 3).

### Methotrexate

#### Mechanism of action

Methotrexate (MTX) acts by irreversibly binding to dihydrofolate reductase, preventing the synthesis of purine and thymidine.^59^ Its anti-inflammatory properties are explained by the intracellular accumulation of 5-amino-1-β-d-ribofuranosyl-imidazole-4-carboxamide (AICAR), leading to an increase in intra- and extracellular adenosine, inhibiting neutrophil chemotaxis and adhesion, superoxide anion formation and secretion of pro-inflammatory cytokines. In addition, MTX becomes polyglutamate intracellularly, and this active metabolite has a much longer duration of action than the original substance,^59^, ^60^ allowing weekly doage with sustained efficacy.^59^, ^61^ It was used for the first time in psoriasis, but it is an effective and safe therapeutic option in several skin diseases, including AD,^62^ dermatomyositis, and lichen planus.^63^

MTX oral absorption is similar to both subcutaneous (SC) and intramuscular (IM) routes, up to a dosage of 15 mg; a superior dosage may lead to a loss of 30%. The SC route shows an increase of absorption proportional to the dosage increase.^64^ MTX has a half-life of 4‒10 h, 50% is bound to circulating proteins and is mostly eliminated by the kidney (70%‒90%), bile, and breast milk.^65^, ^66^ MTX is not mutagenic, although it is abortive and teratogenic, and associated with oligospermia and gynecomastia.^64^, ^65^

The therapeutic dosage varies from 15 to 25 mg/week for adults and 10‒15 mg/m^2^/week for children (oral, intravenous, IM, or SC).^10^, ^11^ The administration of folic acid (1‒5 mg/day: 1 to 6×/week, except on the day of MTX dosage) is recommended to avoid gastric and hematological alterations.^67^

#### Safety

MTX side effects are hematological (low blood counts), gastrointestinal intolerance, renal failure, hepatotoxicity, and, more rarely, acute pneumonitis and pulmonary fibrosis.^59^, ^68^, ^69^, ^70^ Toxicity manifests as mucositis, diarrhea, ulceration of psoriatic plaques, leukopenia, and thrombocytopenia.^59^, ^71^ Folinic acid at a dosage of 10 mg/m^2^ in the initial 24 h is indicated as an antidote to MTX overdose.^72^

#### Evidence

There is a limited number of controlled trials describing MTX in the literature.^11^ A systematic review and meta-analysis showed that the efficacy of MTX was comparable to AZA and lower than dupilumab and ciclosporin at week 16,^14^ with an estimated mean reduction of SCORAD of more than 40% after 12 weeks of treatment in AD patients.^48^ After 24 weeks of treatment with MTX there was a statistically significant reduction in EASI, SCORAD, and pruritus, with an average reduction of 46.7%, 33.6% and 39.1%, respectively, when compared to baseline^62^; therefore, the studies indicate MTX as a moderately effective drug, relatively safe and well-tolerated treatment for moderate-to-severe AD.^11^

The combination of MTX with topical corticosteroids, calcineurin inhibitors or UVB-NB phototherapy is established and considered as safe. Concomitant use of ciclosporin is a relative contraindication. Rheumatoid arthritis experienced a combination of MTX and baricitinib with a safe treatment profile.^11^

#### Monitoring

Before initiating treatment, laboratory tests should be requested: full blood count, liver function and  β-HCG (for women), serology for hepatitis B and C, HIV, PPD and chest X-Ray, according to the target population.^73^ Tests should be repeated 15‒30 days after the initial dosage, with an interval of 1‒3 months, according to medications and comorbidities. In case of persistent alteration of liver function, the drug should be discontinued, and the patient evaluated by a hepatologist.^73^

#### Approval

The use of MTX is off-label for AD treatment in Brazil, but its use is recognized worldwide in international guidelines, consensus, and protocols.^4^, ^13^, ^14^, ^17^

#### Consensus

BSD experts recommended that MTX should be considered as a long-term systemic treatment option for moderate-to-severe AD not responding to topical therapeutic options (Table 3).

### When do we have to consider the need for changes in systemic therapy decision?

The development of novel systemic, targeted-oriented therapies for moderate to severe AD, created a huge demand for an updated and practical guide in Brazil, considering country issues, including coverage of high-cost medication by private insurance or free provision by the local public health system. Internationally a treat-to-target consensus was created which can guide this unmet need.^13^

To summarize, changes in systemic therapy should be based on decision-points such as whether the optimal treatment target was reached after 3 and 6 months. Each decision should take into account improvement in Patient Global Assessment (PGA), plus at least one specific clinical outcome domain.^13^

For clinical disease outcome domains, the initial treatment target goals (after 3 months) should reach at least EASI-50 or SCORAD-50 (50% reduction compared to baseline); peak pruritus NRS (0–10): a reduction of at least 3 points; DLQI: a reduction of at least 4 points; POEM: a reduction of at least 4 points. After 6 months the goal should be to reach at least EASI-75 (75% reduction compared to baseline) or EASI ≤ 7; SCORAD-75 or SCORAD ≤ 24; Peak Pruritus NRS (0–10): an absolute score ≤4; DLQI: an absolute score ≤5; POEM: an absolute score ≤ 7.^13^

According to the patient perspective and targeting patient global assessment, the initial acceptable treatment target goal should reach a reduction of at least 1 point in patient global response (e.g., patient self-reported global assessment of disease severity-PtGA 0‒4) after 3 months, and an absolute score of ≤ 2 after 6 months.^13^

#### Consensus

BSD experts strongly recommend and agree with the treat-to-target consensus,^13^ as described in Table 4, and suggest that these changes in systemic therapy may be also needed in the presence of specific or non-specific undesirable adverse events (e.g., infection) occur under pharmacotherapy; or when there is a contraindication to continuing therapy (e.g., desire to have children; becoming pregnancy).

### Introduction of systemic target-oriented therapies for children and adults

The arsenal of target-specific systemic therapies for treatment of AD is in constant expansion. A recent study presents a systematic review and network meta-analysis, evaluating the standardized mean difference (SMD) of change in the clinical signs of AD. The study allows comparisons of dupilumab and other target-specific medications with conventional systemic drugs for AD using SMD, since there are still no head-to-head studies available.^3^, ^14^

#### Consensus

In accordance with recent international guidelines,^4^, ^11^, ^12^, ^13^, ^14^ BSD experts recommend that when an AD patient is not well controlled with conventional topical and systemic therapies (e.g., phototherapy; CSA or MTX), the introduction of systemic target-oriented therapies approved for moderate-to-severe AD in adults and children such as immunobiologicals and/or Janus-kinase inhibitors should be considered, endorsed by any of the conditions above, except for intention of childbirth; lactating women; and pregnancy (Table 5).

## Systemic target-oriented therapies for adults and children with AD (approved in Brazil)

### Immunobiologicals

#### Dupilumab

##### Mechanism of action

Dupilumab (DUPI) is a recombinant human monoclonal antibody of the IgG4 type that inhibits interleukin-4 and interleukin-13 signaling. Dupilumab inhibits IL-4 signaling through Type I receptor (IL-4R/c) and IL-4 and IL-13 signaling through Type II receptor (IL-4R/IL-13R).^74^

##### Safety

Conjunctivitis is the most common adverse effect (over 30%) mostly mild-to-moderate, without the need to discontinue treatment, and easily manageable with topical anti-inflammatory eyedrops.^11^, ^75^, ^76^, ^77^, ^78^ Injection site reactions, oral herpes simplex infection, and persistent facial erythema are also described as less common adverse events, with no increased risk of eczema herpeticum development under DUPI treatment.^2^, ^79^ Combination therapy of DUPI with topical corticosteroids, topical calcineurin inhibitors, and phototherapy is well established and considered safe.^11^ On-label recommendation includes the avoidance of DUPI concomitant with live virus vaccines.^80^

##### Evidence

###### DUPILUMAB – Adults and adolescents

Phase 3, monotherapy, randomized, double-blind, placebo-controlled studies, 38% (SOLO 1) and 36% (SOLO 2) of adult patients achieved the primary endpoint (IGA 0/1) using DUPI with a first dose of 600 mg SC, followed by 300 mg every 2 weeks, significantly better than placebo (10% and 8% respectively). EASI reduction of 75% and 90% was also significantly better in DUPI groups compared to placebo.^81^ The CHRONOS study evaluated the efficacy over a period of 52 weeks, showing long-lasting improvement rates, as well as maintenance of safety criteria.^82^ The LIBERTY AD CAFÉ study evaluated the efficacy and safety of DUPI 300 mg SC weekly or every 2 weeks plus a concomitant topical corticosteroid in adults with AD and inadequate response or intolerance to CsA. 75% reduction in EASI at week 16 (59.1%, and 62.6% respectively, against 29.6% in the placebo group that used only topical corticosteroids).^83^ A phase 3 randomized clinical trial to assess the efficacy of DUPI monotherapy in adolescents (12‒17 years of age) with moderate to severe inadequately controlled AD resulted in EASI-75 improvement at week 16 in 41.5% every 2 weeks.^84^ A systematic review with meta-analysis showed that compared to DUPI, abrocitinib (200 mg daily), and upadacitinib (30 mg daily), demonstrated associated reductions in EASI scores.^4^ Likewise, the comparison with upadacitinib (15 mg daily), was associated with similar reductions, and tralokinumab (300 mg every other week), and baricitinib (2 and 4 mg daily), were associated with fewer reductions in EASI scores.^4^

###### DUPILUMAB – Pediatric population

A double-blind, 16-week, randomized, phase 3 trial, in children between 6‒11 years, with severe AD, studied the efficacy and safety in concomitant use of 300 mg DUPI every 4 weeks (300 mg Q4W), DUPI every 2 weeks according to weight (< 30 kg ‒ 100 mg Q2W, ≥ 30 kg ‒ 200 mg Q2W), or placebo; with a medium-potency topical corticosteroid. Both groups (Q4W and Q2W DUPI) resulted in clinically meaningful and statistically significant improvement in clinical signs, symptoms, and quality of life versus placebo, as well as a reduction in skin infections. Achieving Investigator Global Assessment (scores of 0 or 1), EASI ≥ 75%, and reduction of itch score were superior in Q4W and Q2W than the placebo group. This study corroborates that the optimal DUPI doses based on efficacy and safety were 300 mg Q4W in children < 30 kg and 200 mg Q2W in children ≥ 30 kg. The most common side effects detected were conjunctivitis and injection-site reactions.^85^

Another double-blind, placebo-controlled, parallel-group, randomized, phase 3 trial was conducted in a pediatric population, aged between 6 months and 6 years of age, with moderate-to-severe AD. DUPI dosage according to bodyweight (≥5 kg to <15 kg: 200 mg; ≥15 kg to <30 kg: 300 mg) every 4 weeks was associated with a low-potency topical corticosteroids ‒ hydrocortisone acetate 1% cream – for 16 weeks. The results confirmed an improvement of the disease in the DUPI group, as well as decreasing associated secondary infections, with comparable results to studies with older children. ^86^

##### Monitoring

Treatment is well tolerated, with no need for screening or follow-up laboratory tests, such as hepatitis serology, HIV and others.^11^, ^75^, ^76^, ^77^, ^78^

##### Approval

DUPI is the first biological therapy for the treatment of AD. It was approved by the Brazilian regulatory agency (ANVISA) in 2017 for use in adults and in 2019 there was extension of use for patients from 12 years of age with moderate to severe AD whose disease is not adequately controlled with topical treatments and systemic immunosuppressants or when these treatments are not recommended, such as in nephropathy or liver disease.^80^ The dosage for adults: initially 600 mg SC day 1 followed by 300 mg Q2W. For 12‒17 years of age: 30 kg to <60 kg: initially 400 mg SC day 1 followed by 200 mg Q2W, and when ≥ 60 kg: initially 600 mg SC day 1 followed by 300 mg Q2W.^47^

Since Aug 12, 2022, DUPI is also approved by ANVISA for use in patients with severe AD aged between 6 months and 11 years of age whose disease is not adequately controlled with topical treatments, or when these treatments are not recommended.^87^ The dosage for the age of 6 months ‒ 5 years: 15 to <30 kg, initial and subsequent dosage of 300 mg Q4W, 1 × 300 mg; 5 to <15 kg, initial and subsequent dosage 200 mg Q4W, 1 × 200 mg).^87^

##### Consensus

BSD experts strongly recommend DUPI for moderate-to-severe AD in adults (above 18 years), adolescents (from 12 to 17 years) and children (from 6 months to 11 years) with refractory disease to topical agents or phototherapy or conventional systemic therapies (e.g., CsA or MTX) (Table 5).

### Janus-kinase inhibitors

The Janus-kinase/signal and activator of transcription (JAK-STAT) pathway is linked to type I/II cytokine receptors.^88^ The JAK family has four members (JAK1, JAK2, JAK3, and TYK2), and when activated, phosphorylation of STAT proteins (7 members) occurs, followed by dimerization and translocation into the nucleus, targeting gene transcription. In AD, the JAK-STAT pathway seems to exert a relevant role in reducing inflammation, pruritus and regulating filaggrin expression^89^, ^90^; IL-4, IL-5, IL-13, IL31, IL-22, and thymic stromal lymphopoietin (TSLP) bind to JAK-STAT–dependent receptors, activating the JAK-STAT cascade (via JAK1-3 and TYK2), therefore upregulating the inflammatory cytokines.^11^, ^91^ Inhibition of the JAK-STAT pathway proved to be an efficacious therapeutic target in inflammatory diseases, and oral JAK inhibitors may exert selective, fast and reversibly blockage of the Th2 cytokine and B-cell mechanisms involved in AD. Approved systemic JAK inhibitors for AD have a high selectivity as follows: anti-JAK1 (Abrocitinib or ABRO; Upadacitinib or UPA); anti-JAK1/2 (Baricitinib or BARI) and anti-JAK1/3 (Tofacitinib or TOFA).^11^, ^88^

The same baseline screening and treatment monitoring is recommended for all JAK inhibitors: full blood count, renal, liver and lipid profile, creatinine phosphokinase levels, hepatitis, and tuberculosis (TB) screening, and chest radiographs.^11^ There must be caution with AD patients older than 65 years and vaccination status; herpes zoster vaccination, when possible, is indicated for patients on JAK inhibitors.^11^

#### Baricitinib

##### Mechanism of action

Baricitinib (BARI) is an oral small-molecule inhibitor of Janus kinase (JAK)1 and JAK2, which have been implicated in the pathogenesis of AD.^11^, ^92^

##### Safety

Nasopharyngitis, folliculitis, oral herpes, upper respiratory tract infection, acne, diarrhea, and back pain were the most frequently reported adverse effects. Seven major adverse cardiovascular events, three pulmonary embolism, and 14 malignancies excluding nonmelanoma skin cancer were described. No deep vein thromboses or tuberculosis were reported.^93^

##### Evidence

Phase III studies of BARI were named BREEZE and evaluated its efficacy in adult patients with moderate to severe AD as monotherapy or in combination with a topical corticosteroid. The doses used in the clinical trials were 1 mg, 2 mg and 4 mg/day. In monotherapy studies, the 4 mg/day dose showed a percentage of patients achieving EASI-75 and -90 of 24.8% and 16.0%, (BREEZE AD-1) and 21.1% and 13% (BREZZE AD-2), respectively.^94^ In the study in combination with topical corticosteroids (BREEZE AD-7), the percentage of patients who reached EASI-75 and -90 was 48% and 24%, respectively, showing that the use of topical corticosteroids potentiates its action.^92^, ^95^ BARI achieved significant and/or clinically relevant improvements in multiple measures of disease severity, pruritus, skin pain, sleep disturbance, and Health-Related Quality of Life (HR-QOL) over 16 weeks and its efficacy generally was sustained over the longer-term treatment duration (≤ 68 weeks).^92^ A network meta-analysis including multiple phase 3 clinical trials, showed a consistent pattern across main clinical outcomes (EASI, POEM, DLQI, PP-NRS), with BARI, 2 and 4 mg daily, associated with slightly worse index scores when compared with DUPI.^4^

##### Monitoring

Full blood count, renal, liver, lipid profiles, and creatinine phosphokinase levels, screening for hepatitis B, C, and HIV, TB screening, including a chest radiograph, PPD or QuantiFERON. Repeat full blood count, renal, liver, and lipid profiles as well as creatinine phosphokinase levels after 4 weeks of treatment and then repeat every three months while on therapy.^11^

##### Approval

Although it was the first JAK inhibitor approved in Brazil for treatment of moderate to severe AD in adults,^96^ its efficacy is lower than DUPI and the other JAK inhibitors.^97^ The recommended dosage for BARI is 4 mg per day, with reduction to 2 mg per day possible, depending on treatment response.

##### Consensus

BSD experts recommend BARI usage for moderate to severe AD for patients who are eligible for systemic therapy and have an inadequate response to other therapies (Table 5).

#### Upadacitinib

##### Mechanism of action

Upadacitinib (UPA) is an oral, highly selective JAK inhibitor, with greater potency against JAK1, with less specificity against JAK2, JAK3, or TYK2.^11^, ^88^, ^89^, ^97^

##### Safety

Blocking the JAK-STAT pathway may lead to hematologic abnormalities such as anemia, neutropenia, or thrombocytopenia. The most common adverse event was acne (15.8%).^98^, ^99^, ^100^ Eczema herpeticum, herpes zoster, and laboratory abnormalities were described in AD patients under UPA therapy.^101^

Reports on the use of UPA with other systemic therapies in AD patients are still rare, but there are reports of combination therapy with MTX (15 mg) in rheumatoid arthritis.

##### Evidence

###### Placebo-controlled studies

UPA has demonstrated superiority against a placebo in controlled studies. One phase 2 trial, including 167 adults, investigated 3 different UPA dosage regimens (30, 15, and 7.5 mg/day over 16 weeks) for AD compared to a placebo. The UPA EASI mean change (SD) was superior to placebo for all dosage groups: 74% (6.1%) for 30 mg, 62% (6.1%) for 15 mg, 39% (6.2%) for 7.5 mg, and 23% (6.4%) for placebo (p = 0.03, <0.001, <0.001, respectively).^102^

Measure Up-1 and Measure Up-2 were two replicate multicenter, randomized, double-blind, placebo-controlled, phase 3 trials that included 1609 patients, adolescents (12‒18 years) and adults (18‒75 years), and compared UPA 30 mg vs. 15 mg vs. placebo once daily for 16 weeks. The co-primary endpoints were EASI-75 and vIGA-AD (validated investigator global assessment for atopic dermatitis) score of 0 or 1. In Measure Up-1/2, EASI-75 was present in 71.3%/60.4% (UPA 15 mg), 81.6%/74.4% (UPA 15 mg) and 17.1%/13.9% (placebo). vIGA 0 or 1 was achieved in 49.5%/39.3% (UPA 15 mg); 64.4%/53.4% (UPA 30 mg) and 9.3%/5.2% (placebo).^98^ During extended follow-up of Measure Up-1 and -2 until week 52, there was sustained efficacy: EASI-75 was achieved at 69%/68.8% (15 mg) and 74.5%/71.5% (30 mg).^99^ Studies with UPA in combination with corticosteroids showed similar results to Measure Up-1 and -2.^103^

###### UPA and DUPI

The Heads-Up study compared adults with severe AD in the use of UPA 30 mg orally once daily (up to week 24) versus SC DUPI 300 mg Q2W (600 mg loading dosage, starting at week 2 and until week 22). The primary endpoint was EASI-75 in week 16. UPA (n = 348) was superior to DUPI (n = 344) (71% vs. 61.1% at week 16 [p = 0.006]) and demonstrated superiority in secondary endpoints (EASI-90, EASI-100, and pruritus improvement). After 16 weeks, 245 patients switched to UPA and enrolled in an OLE study. Patients who did not achieve EASI-75 with DUPI and switched to UPA achieved this score at week 4 (75%) and at week 16 (87.5%), respectively, after switching to UPA.^100^

A meta-analysis demonstrated that UPA (30 mg daily) was more related to reduced EASI than DUPI (600 mg initially and then 300 mg every 2 weeks) up to 16 weeks of treatment in adults with AD.^4^

##### Monitoring

Prior to treatment full blood count, renal, liver, and lipid profiles, and creatinine phosphokinase levels, screening for hepatitis B, C, and HIV, TB screening, including a chest radiograph, PPD or QuantiFERON.^11^

During UPA therapy: repeat full blood count, renal, liver, and lipid profiles as well as creatinine phosphokinase levels after 4 weeks of treatment and then repeat every three months while on therapy.^11^

##### Approval

UPA has been approved to treat severe AD in patients above 12 years^76^ in the USA, Europe, UK, Japan^89^ and Brazil (ANVISA, 2022).^104^

##### Consensus

BSD experts recommend UPA usage for moderate to severe AD who are eligible for systemic therapy (Table 5).

#### Abrocitinib

##### Mechanism of action

Abrocitinib (ABRO) is an oral, selective, and potent inhibitor of Janus kinase-1 (JAK1), with a recommended dosage of 100 or 200 mg once daily.^91^ The mechanism of action by inhibiting the JAK1 pathway modulates multiple cytokines involved in the pathophysiology of AD, including IL-4, IL-13, IL-31, IL-22, and IFN-γ and TSLP.^91^, ^105^

##### Safety

The main adverse events related to ABRO were nausea (200 mg/day), and nasopharyngitis and AD exacerbation (100 mg/day), when compared with placebo.^106^, ^107^ Other adverse events dosage-related were headaches, acne, and an increase in herpes simplex and herpes zoster infections.^11^

##### Evidence

Phase 2 studies have shown favorable results with dosages of 100 mg and 200 mg, with a reduction of disease severity and pruritus.^108^ Phase 3 studies called JADE evaluated patients with moderate and severe AD with different objectives. JADE MONO-1 and JADE MONO-2 ‒ ABRO 100 and 200 mg compared to placebo in adults and adolescents over 12 years of age. ABRO was more effective than placebo, better with a dosage of 200 mg. Rapid reduction of pruritus after 24 hours of the first dose. JADE TEEN – ABRO 100 mg, 200 mg and placebo combined with topical therapy (corticosteroid, calcineurin inhibitor, or phosphodiesterase-4 inhibitor) in adolescents aged 12 to 17 years, showed efficacy and safety similar to MONO-1 and -2 studies.^109^ JADE REGIMEN evaluated maintenance therapy in adults and adolescents over 12 years of age. A study with an open induction phase with ABRO 200 mg in monotherapy for 12 weeks was conducted; at the maintenance phase, AD patients were randomized to ABRO 200 mg, or dose reduction to 100 mg or placebo, for 40 weeks, indicating that treatment induction with ABRO 200 mg followed by 100 mg may be a viable strategy. There were fewer adverse events with ABRO 100 mg compared to 200 mg at maintenance.^110^ JADE EXTEND analyzed the extension phase, with the objective of evaluating the efficacy and safety of ABRO 100 or 200 mg in patients who had already used DUPI. It showed good results even in patients with previous use of the immunobiological.^111^ In conclusion, these studies (JADE) showed that ABRO is effective and safe to treat moderate to severe AD.

##### Monitoring

The same baseline screening is recommended for all JAK inhibitors: full blood count, renal, liver and lipid profiles as well as creatinine phosphokinase levels and hepatitis and TB screening, including a chest radiograph.^11^ The recommendation for monitoring is full blood count, renal, liver and lipid profiles as well as creatinine phosphokinase level at 4 weeks after starting treatment and then every three months while on ABRO therapy.^11^

##### Approval

In September 2021, ABRO received approval in the UK and Japan for treatment of adolescents (> 12 years) and adults with moderate to severe AD, eligible for systemic therapy and with inadequate response to other therapies. In December 2021, it was approved in the European Union for adults; in January 2022 by the FDA (USA) for the treatment of moderate to severe refractory AD over 18 years, and in February 2023 for adolescents and teens (from 12‒17 years).^11^, ^112^, ^113^ In Brazil, it was approved in June 2023 for adolescents (> 12 years) and adults with moderate to severe AD.^114^

##### Consensus

In summary, ABRO at a starting dosage of 200 mg once daily is recommended for adults with AD. After a satisfactory response, the dosage can be reduced to 100 mg daily. In patients aged ≥ 65 years and adolescents, a starting dose of 100 mg once daily is recommended.^4^ A recent trial showed better results with ABRO 200 mg daily compared to DUPI.^115^

BSD experts endorse ABRO usage for moderate to severe AD who are eligible for systemic therapy and have an inadequate response to other therapies equate response to other therapies (Table 5).

### Systemic target-therapies for AD patients (underanalysis for license in Brazil)

#### Tralokinumab

##### Mechanism of action

Tralokinumab (TRALO) is a fully human monoclonal antibody of the IgG4 type that specifically binds to IL-13 and inhibits its interaction with IL-13 receptors.^116^, ^117^

##### Safety

The main adverse events were upper airway infection and conjunctivitis (notably less than with the use of DUPI).^118^

##### Evidence

In phase 3 studies (ECZTRA) adult patients with moderate and severe AD were on TRALO monotherapy 300 mg SC Q2W, compared to placebo.^118^, ^119^ The drug was more effective than the placebo with reduced pruritus, improved sleep quality, improved quality of life, and disease severity scores. Outcomes were IGA 0 or 1, EASI-75 at week 16, and most responders maintained good responses at week 52.^118^

TRALO has been tested with concomitant use of topical corticosteroids. Adults with moderate to severe AD achieved IGA 0/1 and EASI-75; better results than the placebo group. Ninety percent of responders sustained a response in week 32.^119^

##### Monitoring

Laboratory monitoring is not required.^119^

##### Approval

TRALO obtained approval for treatment of moderate to severe AD on June 22^nd,^ 2021, by the EMA in Europe and on December 27 of the same year by the FDA (USA). In Brazil, it awaits licensing.

##### Consensus

The recommended starting dosage of TRALO for patients aged 12 years and older is 600 mg (four 150 mg injections) and its recommended maintenance dosage is 300 mg (two 150 mg injections) given every other week.^11^ At the discretion of the prescriber, a dosage every four weeks may be considered for patients who achieved clear or nearly clear skin after 16 weeks of treatment.^11^

BSDs experts endorse TRALO usage for moderate to severe AD who are eligible for systemic therapy and have an inadequate response to other therapies, after approval by the Brazilian regulatory agency.

**Figure 1:**
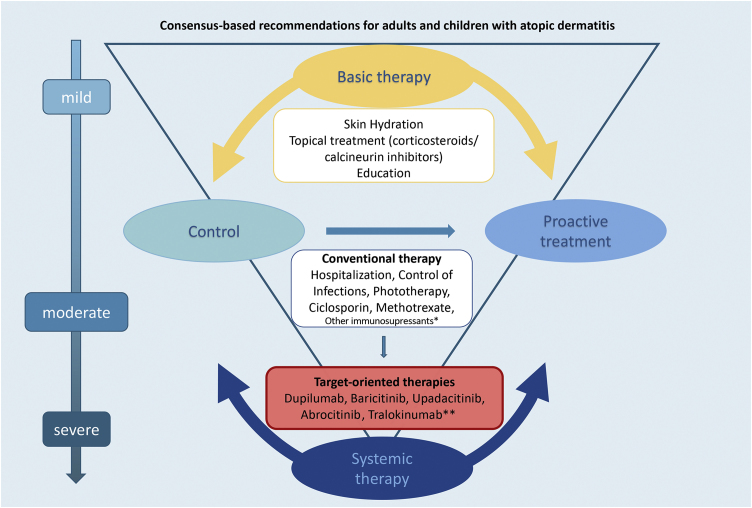
Basic and systemic AD therapy: overview of SBD expert consensus-based recommendations for adults and children. *Other immunosuppressants: azathioprine and mycophenolate mofetil. ** Awaiting approval in Brazil

Fig. 1 summarizes the basic and systemic therapeutic options for AD in children and adults discussed in this updated consensus.

The authors believe that this study will contribute to establishing practical indications for the use and prescription of phototherapy and systemic therapies for AD patients, including practical adequate therapeutic modalities such as phototherapy and systemic therapies for AD patients, adding core outcome instruments to unify outcome reports in clinical trials and clinical practice for Brazilian physicians. Limitations of use include different age groups, ethnic-racial variations, or accessibility to either conventional or target-specific therapies. These factors should be taken into account when indicating systemic treatment options for AD (Fig. 1).

## Financial support

Brazilian Society of Dermatology.

## Authors' contributions

### Project coordinators

Raquel Leao Orfali: Project coordination; conception and design of the study; data collection; statistical analysis; article writing and critical review of the content; obtaining, analyzing, and interpreting data; critical review of the literature; final approval of the final version of the manuscript.

Valeria Aoki: Project coordination; conception and design of the study; data collection; statistical analysis; article writing and critical review of the content; obtaining, analyzing, and interpreting data; critical review of the literature; final approval of the final version of the manuscript.

Daniel Lorenzini: Project coordination; conception and design of the study; data collection; statistical analysis; article writing and critical review of the content; obtaining, analyzing, and interpreting data; critical review of the literature; final approval of the final version of the manuscript.

Phyllis I. Spuls: Conception and design of the study; critical review of the content; critical review of the literature; final approval of the final version of the manuscript.

Louise A. A. Gerbens: Conception and design of the study; critical review of the content; critical review of the literature; final approval of the final version of the manuscript.

Authorship contribution by the other participating Brazilian experts

Aline Bressan: Article writing and critical review of the content; final approval of the final version of the manuscript.

Anber Ancel Tanaka: Article writing and critical review of the content; final approval of the final version of the manuscript.

Ana Maria Mósca de Cerqueira: Article writing and critical review of the content; final approval of the final version of the manuscript.

André da Silva Hirayama: Article writing and critical review of the content; final approval of the final version of the manuscript.

Andréa Machado Coelho Ramos: Article writing and critical review of the content; final approval of the final version of the manuscript.

Carolina Contin Proença: Article writing and critical review of the content; final approval of the final version of the manuscript.

Claudia Marcia de Resende Silva: Article writing and critical review of the content; final approval of the final version of the manuscript.

Cristina Marta Maria Laczynski: Article writing and critical review of the content; final approval of the final version of the manuscript.

Francisca Regina Carneiro: Critical review of the content; final approval of the final version of the manuscript.

Gleison Duarte: Article writing and critical review of the content; final approval of the final version of the manuscript.

Gunter Hans Filho: Article writing and critical review of the content; final approval of the final version of the manuscript.

Heitor de Sá Gonçalves: Critical review of the content; final approval of the final version of the manuscript.

Ligia Pessoa de Melo: Critical review of the content; final approval of the final version of the manuscript.

Luna Azulay-Abulafia: Critical review of the content; final approval of the final version of the manuscript.

Magda Blessmann Weber: Article writing and critical review of the content; final approval of the final version of the manuscript.

Maria Cecília Rivitti-Machado: Article writing and critical review of the content; final approval of the final version of the manuscript.

Mariana Colombini Zaniboni: Article writing and critical review of the content; final approval of the final version of the manuscript.

Marília Ogawa: Article writing and critical review of the content; final approval of the final version of the manuscript.

Mario Cezar Pires: Article writing and critical review of the content; final approval of the final version of the manuscript.

Mayra Ianhez: Article writing and critical review of the content; final approval of the final version of the manuscript.

Paulo Antonio Oldani Felix: Article writing and critical review of the content; final approval of the final version of the manuscript.

Renan Bonamigo: Critical review of the content; final approval of the final version of the manuscript.

Roberto Takaoka: Article writing and critical review of the content; final approval of the final version of the manuscript.

Rosana Lazzarini: Article writing and critical review of the content; final approval of the final version of the manuscript.

Silmara Cestari: Article writing and critical review of the content; final approval of the final version of the manuscript.

Silvia Assumpção Soutto Mayor: Article writing and critical review of the content; final approval of the final version of the manuscript.

Tania Cestari: Article writing and critical review of the content; final approval of the final version of the manuscript.

Zilda Najjar Prado de Oliveira: Critical review of the content; final approval of the final version of the manuscript.

## Conflicts of interest

Raquel Leao Orfali: Participation in Sanofi and Lilly clinical trials, and as consultant to Bayer and Abbvie.

Daniel Lorenzini: Participation in Lilly clinical trials, consultant to Sanofi, Abbvie and Pfizer.

Valeria Aoki: Participation in Sanofi and Lilly clinical trials, and as consultant to Abbvie and Pfizer.

Phyllis I. Spuls: Received departmental independent research grants from pharmaceutical industries since December 2019 for the TREAT NL registry, is involved in performing clinical trials with many pharmaceutical industries that manufacture drugs used for the treatment of e.g. psoriasis and atopic dermatitis, for which financial compensation is paid to the department/hospital and, is Chief Investigator (CI) of the TREatment of ATopic eczema (TREAT) national registry (TREAT NL) and international taskforce (TREAT Registry Taskforce) on photo- and systemic therapy in adults and children.

Louise A. A. Gerbens: Member of the European guideline (EuroGuiDerm) on atopic dermatitis, member of the Dutch guideline on atopic dermatitis, one of the (chief) investigators of the TREatment of ATopic eczema (TREAT) national registry (TREAT NL) and international taskforce (TREAT Registry Taskforce) on photo- and systemic therapy in adults and children.

Aline Bressan: Speaker for Janssen and Abbvie.

Anber Ancel Tanaka: Consulting and speaker for Abbvie, Lilly, Janssen, Sanofi, Novartis, Leo Pharma.

Ana Maria Mósca de Cerqueira: Advisory board for L’Óreal, Sanofi, Pierre-Fabre, Medihealth, Pfizer, and Lilly; Speaker for L’Óreal, Mustela, Theraskin, Pfizer, Conatec, Aché, Bagó and Johnson & Johnson.

André da Silva Hirayama: Advisory board and speaker for Janssen, Abbot/Abbvie, Leo Pharma, Boehringer Ingelheim, Novartis, Lilly; partition in clinical trials for Janssen, Abbot/Abbvie, Boehringer Ingelheim, Novartis, Roche, Lilly, Allergan.

Andréa Machado Coelho Ramos: None.

Carolina Contin Proença: None.

Claudia Marcia de Resende Silva: None.

Cristina Marta Maria Laczynski: Advisory board and speaker for Abbvie, Leo Pharma, and Libbs; participation in clinical trials for Lilly; participation in clinical trials and speaker for Pfizer; speaker for Sanofi and Theraskin.

Francisca Regina Carneiro: None.

Gleison Duarte: Advisory board and speaker for Abbvie, Lilly, Janssen, Galderma, Sanofi, Novartis, UCB, Leo Pharma. Participation in Amgen, Novartis, and Sanofi clinical trial.

Gunter Hans Filho: Participation in clinical trial for Sanofi, and Principia Biopharma; speaker for Galderma.

Heitor de Sá Gonçalves: None

Ligia Pessoa de Melo: Advisory board and speaker for Galderma, Sanofi and Abbvie.

Luna Azulay-Abulafia: Adboard for Galderma; clinical trials for Pfizer and Lilly.

Magda Blessmann Weber: Speaker for Lilly and Abbvie; clinical researcher for Lilly and Sanofi.

Maria Cecília Rivitti-Machado: Adboard, consulting and speaker for Abbvie, Novartis, Pfizer, Sanofi, Janssen, Mantecorp.

Mariana Colombini Zaniboni: None.

Marília Marufuji Ogawa: None.

Mario Cezar Pires: Adboard, consulting and speaker for AbbVie, Sanofi, Pfizer, Janssen-Cilag, Ely-Lilly, Novartis, UCB, LeoPharma, Amgen, Sandoz.

Mayra Ianhez: Participation as a speaker for Sanofi, Abbvie, Janssen, Novartis, Galderma, Theraskin, Pfizer and in advisory boards for Sanofi, Abbvie, Jannsen, Novartis and Galderma.

Paulo Antônio Oldani Felix: Consultant and speaker for AbbVie, Sanofi, Pfizer, Janssen-Cilag, Ely-Lilly, Novartis, UCB, LeoPharma, Amgen, Sandoz.

Renan Bonamigo: None.

Roberto Takaoka: Consultant to Pfizer and Johnson & Johnson.

Rosana Lazzarini: None.

Silmara Cestari: Advisory board and speaker for Abbvie, Biolab, Grupo Loreal, Johnson & Johnson, Leo Pharma, Libbs, Mustela-Expanciense, Sanofi, Pfizer.

Silvia Assumpção Soutto Mayor: Adboard for Sanofi and speaker for Abbvie, Pfizer, Sanofi, Libbs, Mustela-Expanciense

Tania Cestari: None.

Zilda Najjar Prado de Oliveira: None.
